# Interleukin-1β Modulation of the Mechanobiology of Primary Human Pulmonary Fibroblasts: Potential Implications in Lung Repair

**DOI:** 10.3390/ijms21228417

**Published:** 2020-11-10

**Authors:** Marta Gabasa, Marselina Arshakyan, Alejandro Llorente, Lourdes Chuliá-Peris, Irina Pavelescu, Antoni Xaubet, Javier Pereda, Jordi Alcaraz

**Affiliations:** 1 Unit of Biophysics and Bioengineering, Department of Biomedicine, School of Medicine, Universitat de Barcelona, 08036 Barcelona, Spain; aeryn13@gmail.com (M.G.); m.arshakyan@ub.edu (M.A.); alejandro.llorente.alvarez@gmail.com (A.L.); iripavelescu@gmail.com (I.P.); 2 Departament of Physiology, Faculty of Pharmacy, Universitat de València, 46100 València, Spain; lourdes.chulia@uv.es (L.C.-P.); javier.pereda@uv.es (J.P.); 3 Pneumology Service, Hospital Clínic, 08036 Barcelona, Spain; axaubet@clinic.ub.es; 4 CIBER de Enfermedades Respiratorias (CIBERES), 28029 Madrid, Spain; 5 Institute for Bioengineering of Catalonia (IBEC), The Barcelona Institute for Science and Technology (BIST), 08028 Barcelona, Spain

**Keywords:** IL-1β, pulmonary fibroblasts, repair, cell mechanics, collagen, MMPs

## Abstract

Pro-inflammatory cytokines like interleukin-1β (IL-1β) are upregulated during early responses to tissue damage and are expected to transiently compromise the mechanical microenvironment. Fibroblasts are key regulators of tissue mechanics in the lungs and other organs. However, the effects of IL-1β on fibroblast mechanics and functions remain unclear. Here we treated human pulmonary fibroblasts from control donors with IL-1β and used Atomic Force Microscopy to unveil that IL-1β significantly reduces the stiffness of fibroblasts concomitantly with a downregulation of filamentous actin (F-actin) and alpha-smooth muscle (α-SMA). Likewise, *COL1A1* mRNA was reduced, whereas that of collagenases *MMP1* and *MMP2* were upregulated, favoring a reduction of type-I collagen. These mechanobiology changes were functionally associated with reduced proliferation and enhanced migration upon IL-1β stimulation, which could facilitate lung repair by drawing fibroblasts to sites of tissue damage. Our observations reveal that IL-1β may reduce local tissue rigidity by acting both intracellularly and extracellularly through the downregulation of fibroblast contractility and type I collagen deposition, respectively. These IL-1β-dependent mechanical effects may enhance lung repair further by locally increasing pulmonary tissue compliance to preserve normal lung distension and function. Moreover, our results support that IL-1β provides innate anti-fibrotic protection that may be relevant during the early stages of lung repair.

## 1. Introduction

There is a growing awareness that each tissue has unique mechanical properties and that such properties are essential to support tissue-specific functions [1,2]. In the context of normal lung physiology, pulmonary tissue remains soft and elastic to accommodate the cyclic volume changes required for breathing [3,4]. This normal tissue elasticity is transiently compromised in response to local tissue damage to facilitate repair [5,6,7,8]. In contrast, failure to resolve injury and restore normal lung architecture and function occurs concomitantly with persistent alterations in pulmonary tissue elasticity in a variety of physiopathological conditions, eliciting either a marked stiffening (as in pulmonary fibrosis or lung cancer) or softening (as in emphysema) [3,9,10,11]. Accordingly, there is increasing interest in identifying key mechanistic aspects of the mechanobiology underlying normal tissue homeostasis and repair and how it becomes awry in disease conditions in the lung and other organs, with the ultimate goal to develop therapies that restore the normal tissue mechanical microenvironment and function [5].

The elasticity of lungs and other soft tissues is determined by the mechanical characteristics of cells and their surrounding extracellular matrix (ECM) as well as by the bidirectional cell-ECM mechanical interactions [3,4,12,13]. Following damage, tissue elasticity becomes temporarily disrupted as part of the normal tissue host response. Fibroblast rank among the stiffest pulmonary cell types [13,14,15] and have been identified as key regulators of tissue mechanics during repair as well as in cell-biomaterial responses owing to their unique ability to rapidly alter tissue contractility and efficiently remodel the ECM in response to reparatory cytokines [6,8,16]. Despite recent efforts towards dissecting the biological complexity underlying tissue repair in the lung and other organs [8,16], our overall understanding is still scarce. Moreover, from the mechanical standpoint, most previous studies on fibroblast have focused on their responses upon activation by transforming growth factor-beta (TGF-β), which becomes upregulated either transiently at the late stages of canonical tissue repair or chronically during fibrosis and cancer in the lung and other organs [8,17,18]. Indeed, we and others have consistently reported a marked fibroblast stiffening in response to TGF-β and have identified key underlying signaling events [15,19,20]. In contrast, little is known on the mechanobiology alterations of fibroblasts upon stimulation with inflammatory cytokines like interleukin-1β (IL-1β), as it occurs during earlier stages of tissue response to damage [6,7,8].

Following tissue injury, damaged cells first release inflammatory factors that draw an influx of immune cells like neutrophils and monocytes [16]. IL-1β is one of the most well-studied members of the important IL-1 family of proteins that are key regulators of inflammation and tissue homeostasis in the lung and other organs [21,22,23]. IL-1β is primarily expressed by monocytes and macrophages stimulated by pathogen products or factors released by damaged cells, whereas it is not generally expressed in healthy tissue cells [22,24]. The effects of IL-1β contribute to the regulation of local inflammatory and repair responses, and its dysregulation has been implicated in numerous lung physiopathological conditions, including pulmonary fibrosis, asbestosis, chronic obstructive pulmonary disease (COPD), emphysema, severe cases of COVID-19, and other types of acute lung injury [23,24,25,26,27], which have drawn the interest of the pharmaceutical industry [23]. However, while there is a clear consensus that TGF-β stiffens fibroblasts [17], such consensus is missing on the direct mechanical impact of IL-1β on fibroblasts or other stromal cells since the few studies available on this topic have reported conflicting results [19,22,28,29]. Likewise, it is unclear the impact of IL-1β on ECM deposition by fibroblasts, particularly on fibrillar collagens [24,30], which could further alter tissue mechanics.

The simplest and most widely used physical parameter to characterize the mechanical properties of cells and other biological samples is Young’s elastic modulus (*E*), which quantifies sample resistance to deformation [13]. Atomic force microscopy (AFM) has been extensively used to measure *E* locally in cells at the nanometer-length scale. For this purpose, AFM uses a nanometer-sized tip at the end of a flexible cantilever (the force sensor) to gently apply compressive forces on the cell while monitoring its local deformation or indentation [13,31]. We and others have used AFM to probe the mechanics of fibroblasts and other cell types cultured in normal-like and disease-like conditions, including TGF-β stimulation [15,31,32,33,34]. In contrast, AFM has not yet been used to probe fibroblast mechanics in response to pro-inflammatory factors like IL-1β. The goal of this work was to examine the impact of the pro-inflammatory cytokine IL-1β on the mechanics of human pulmonary fibroblasts by AFM as well as their collagen deposition and to assess its potential effects in fibroblast-associated repair processes.

## 2. Results

### 2.1. Primary Human Pulmonary Fibroblasts Exhibit a Marked Stiffness Reduction upon Stimulation with the Pro-Inflammatory Cytokine IL-1β

**Figure 1:**
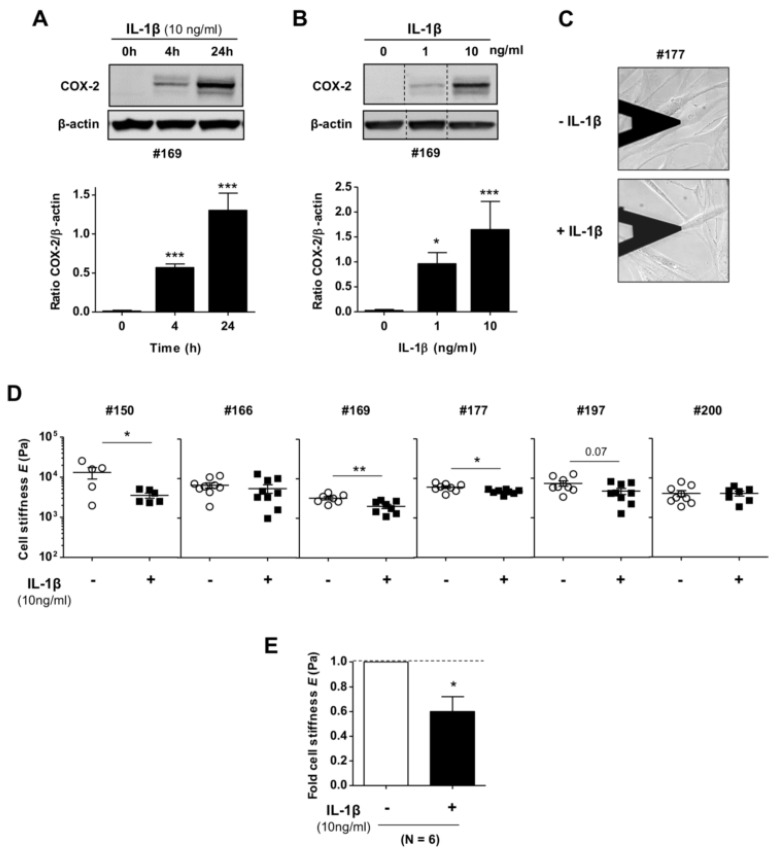
Effect of interleukin-1β (IL-1β) stimulation on the stiffness of primary human pulmonary fibroblasts. (**A**,**B**) Representative western blot of COX-2 and β-actin of fibroblasts stimulated with IL-1β at different time points A (top) and doses B (top); (bottom panels) corresponding densitometric analysis. (**C**) Representative phase contrast image of an atomic force microscopy (AFM) force sensor (cantilever) on top of a single fibroblast stimulated with or without IL-1β for three days. The tip located at the end of the cantilever was used to indent the perinuclear region of each cell locally while monitoring the corresponding opposing force to compute Young´s modulus (*E*). (**D**) Total *E* values obtained on single fibroblasts from our donor cohort (identified by the symbol #150–200) upon IL-1β stimulation as in (**B**). (**E**) Fold average *E* of single fibroblasts measured from each donor shown in **C**. Horizontal dashed line corresponds to no change (fold ratio = 1). * *p* < 0.05, ** *p* < 0.01, *** *p* < 0.005 were determined by Student’s t-test (here and thereafter) comparing either different time-points with respect to 0 h (**A**) or presence and absence of IL-1 β stimulation (**B**,**D**,**E**).

A major downstream signaling event elicited by the pro-inflammatory cytokine IL-1β is the overexpression of cyclooxygenase-2 (COX-2) [35]. To analyze the effects of IL-1β on the mechanobiology of pulmonary fibroblasts, we benefited from our previous work, which identified treating fibroblasts with 10 ng/mL IL-1β for either 24 h or 72 h as sufficient to achieve a robust activation of either COX-2 or the extended COX pathway, respectively [35,36]. We confirmed the suitability of the latter IL-1β timing and dose by analyzing COX-2 expression by Western blot in pulmonary fibroblasts from a randomly selected patient from our cohort, which revealed a consistent time-dependent (Figure 1A) and dose-dependent (Figure 1B) increase in COX-2. Accordingly, we used 10 ng/mL IL-1β thereafter. Such an increase in COX-2 elicited by IL-1β did not compromise the typical elongated spindle-like morphology observed in untreated control conditions (Figure 1C). In contrast, AFM nanoindentation measurements revealed that stimulating control pulmonary fibroblasts with IL-1β for three days consistently reduced the average Young’s elastic moduli (*E*) of cells from most of the randomly selected donors from our cohort (*n* = 6) (Figure 1D), eliciting in average a significant ~40% reduction in *E* compared to untreated cells (Figure 1E). The clinical characteristics of our donor cohort are shown in Table 1. These results revealed that IL-1β alone was sufficient to markedly attenuate the stiffness of healthy human pulmonary fibroblasts.

### 2.2. IL-1β-Dependent Fibroblast Softening Is Associated with a Reduction of Actin Stress Fibers and Expression of α-SMA

**Figure 2:**
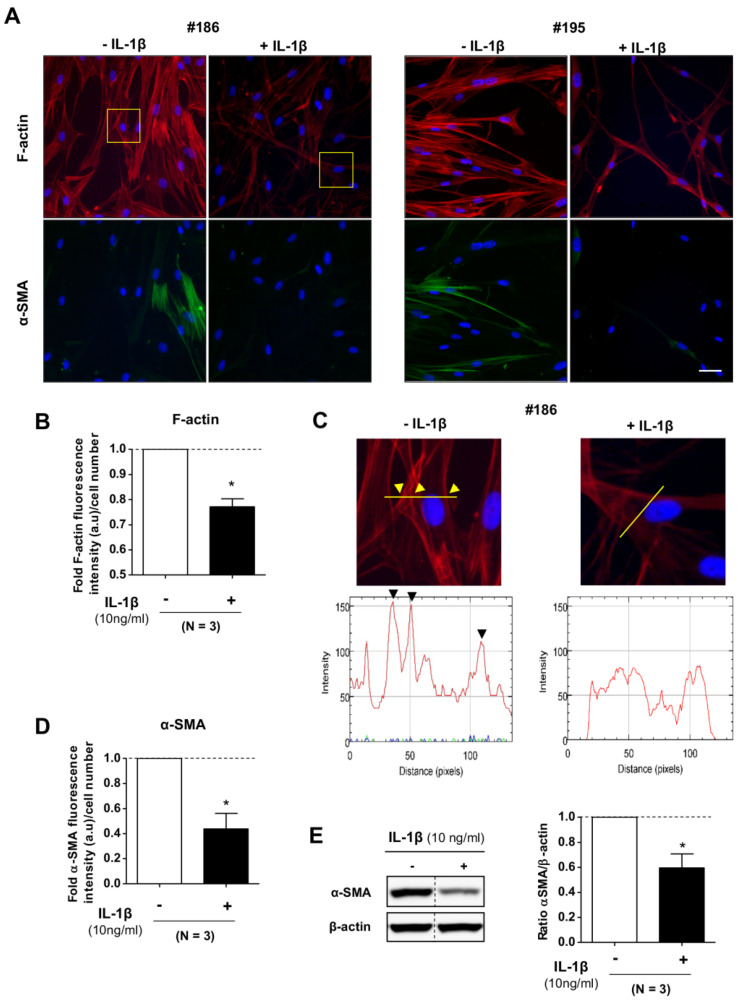
Immunofluorescence analysis of the actomyosin cytoskeleton of primary pulmonary fibroblasts upon stimulation with 10 ng/mL IL-1β. (**A**) Representative immunofluorescence images of filamentous actin (F-actin) (red), alpha-smooth muscle (α-SMA) (green), and DAPI (blue) with or without IL-1β in fibroblasts from two randomly selected donors (#186, #195). Scale bar, 50 μm. (**B**) Fold average F-actin intensity/image normalized by the corresponding cell number in fibroblasts from our cohort (*n* = 3). (**C**) Zoom of the F-actin staining area labeled by a yellow square in A (top); (bottom) quantification of the F-actin fluorescence intensity across the yellow line drawn in the top images to illustrate the loss of stress fibers (seen as intensity peaks) upon stimulation with IL-1β. Arrowheads point at stress fibers and their corresponding fluorescence intensity peak. (**D**) Fold average α-SMA intensity/image normalized by the corresponding cell number in fibroblasts from our cohort (*n* = 3). (**E**) Representative western blot of α-SMA and β-actin at 24 h with or without IL-1β (10 ng/mL) in fibroblasts from a randomly selected donor (left); (right) corresponding densitometry analysis from fibroblasts from our cohort (*n* = 3). Statistical analysis as in Figure 1, * *p* <0.05.

A large body of work has identified the actin cytoskeleton (CSK) as an essential regulator of cell mechanics in fibroblasts and other cell types [13,32,37,38]. Prompted by the marked reduction of *E* elicited by IL-1β, we wondered if such reduction was mediated through changes in the actin CSK. To examine this possibility, we stained filamentous actin (F-actin) as well as alpha-smooth muscle (α-SMA), which is a standard marker of fibroblast activation known to increase fibroblast contractility [15,20]. Immunofluorescence imaging revealed that IL-1β consistently altered both the quantity and organization of F-actin in pulmonary fibroblasts randomly selected from our donor cohort (*n* = 3) (Figure 2A). Specifically, IL-1β elicited a drop in the amount of F-actin staining per cell (Figure 2B) concomitantly with a major reduction in the number of F-actin-rich stress fibers (Figure 2C), which is a cytoskeletal structure strongly associated with cell stiffness [37]. Likewise, even though the expression of α-SMA was limited to few fibroblasts in control conditions (Figure 2A), IL-1β attenuated α-SMA expression, as shown by immunofluorescence (Figure 2D) and Western blotting (Figure 2E). Collectively, these results reveal that IL-1β profoundly remodeled the actomyosin CSK by reducing the number of F-actin-rich stress fibers and the expression of the contractile marker α-SMA.

### 2.3. IL-1β Favors a Reduction in Type I Collagen and Increases the Ratio of COL3A1/COL1A1

**Figure 3:**
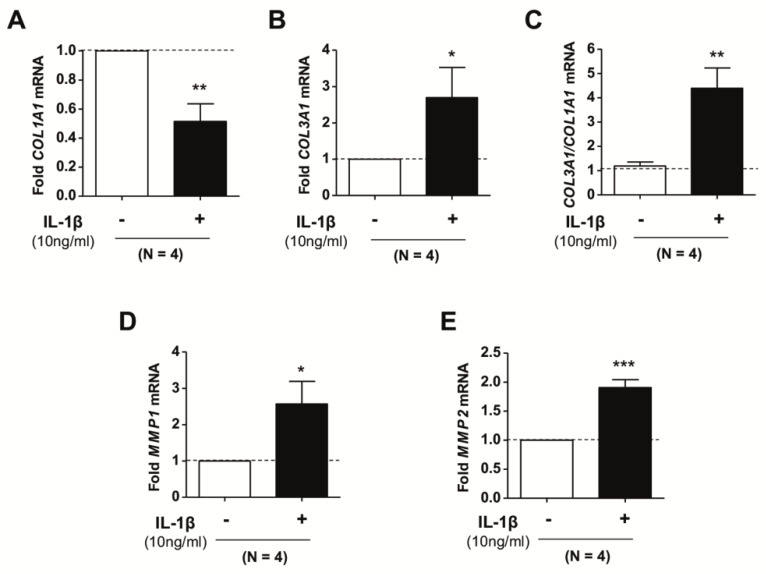
Expression of fibrillar collagens and major collagenases in primary pulmonary fibroblasts upon stimulation with 10 ng/mL IL-1β for 72 h assessed by qRT-PCR. (**A**,**B**) mRNA levels of *COL1A1* (**A**) and *COL3A1* (**B**) in fibroblasts from randomly selected donors (*n* = 4). (**C**) Corresponding *COL3A1/COL1A1* ratio. (**D**,**E**) mRNA levels of *MMP1* (**D**) and *MMP2* (**E**). Statistical analysis as in Figure 1, * *p* < 0.05, ** *p* < 0.01, *** *p* < 0.005.

The collagen family of ECM components are abundantly expressed in the lungs and other organs and tissues and are known to play a major role in tissue mechanics [39]. Fibrillar collagens, including type I and type III collagen—whose transcription is partly encoded by *COL1A1* and *COL3A1* genes, respectively—are particularly relevant in regulating tissue mechanical strength and are highly abundant in the pulmonary interstitium [12]. To assess the impact of IL-1β on the expression of fibrillar collagens, we stimulated pulmonary fibroblasts randomly selected from our donor cohort (*n* = 4) with IL-1β for three days and examined the expression of *COL1A1* and *COL3A1* by qRT-PCR. We observed that IL-1β induced a significant reduction in *COL1A1* (Figure 3A) concomitantly with a significant increase in *COL3A1* (Figure 3B) mRNA (Supplementary Figure S1), thereby eliciting a marked increase in the *COL3A1*/*COL1A1* ratio (Figure 3C), which has been previously associated with scar-less repair [40]. In addition, since the turnover of fibrillar collagens is controlled not only by the expression of *COL1A1* and *COL3A1* but also by that of their degrading collagenolytic enzymes, we examined the impact of IL-1β on matrix metalloproteinases 1 and 2 (*MMP1* and *MMP2*), which are two major secreted collagenases [41]. Our results revealed that IL-1β significantly increased both *MMP1* and *MMP2* mRNA in pulmonary fibroblasts compared to untreated cells (Figure 3D,E). These results support that IL-1β favors a reduction of type I collagen in human pulmonary fibroblasts by downregulating *COL1A1* mRNA while increasing mRNA levels of major collagenolytic enzymes.

### 2.4. Pulmonary Fibroblasts Exhibit an Increased Migration yet an Attenuated Proliferation and Ability to Close the Gap in a Scratch Wound Assay upon Stimulation with IL-1β

**Figure 4:**
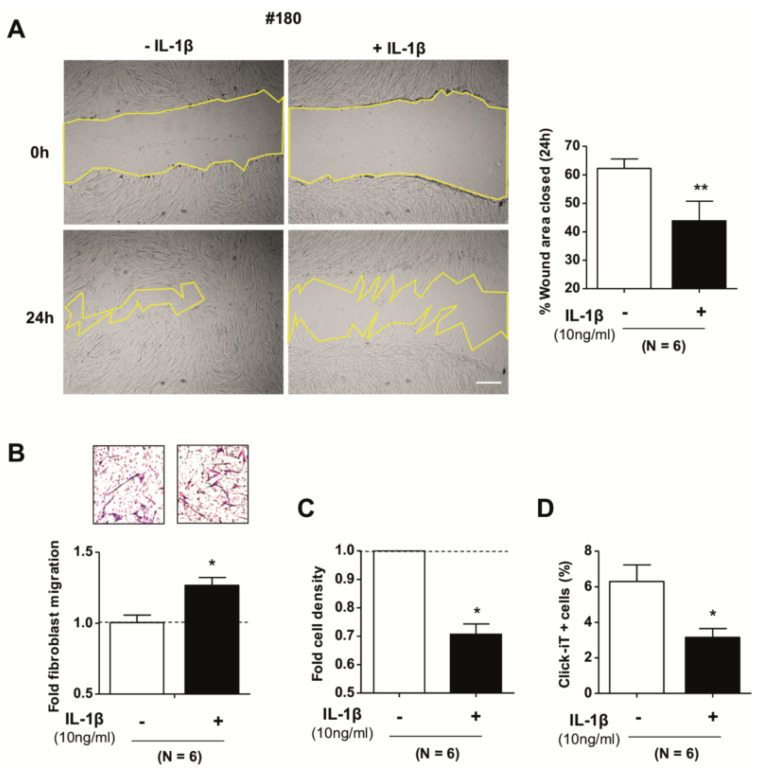

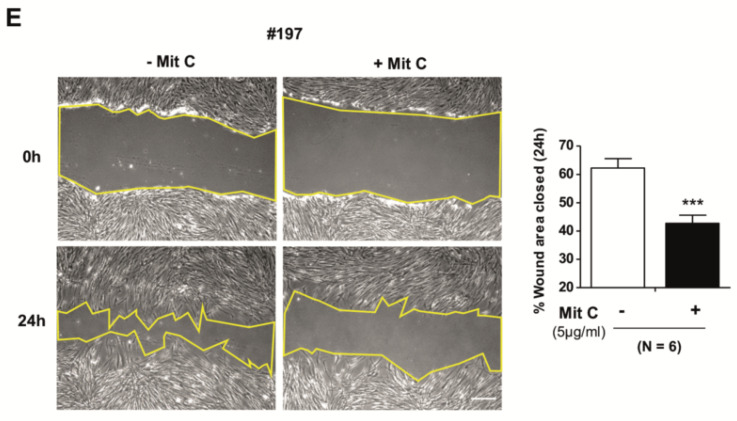
Effect of IL-1β on repair-associated processes in primary pulmonary fibroblasts. (**A**) An in vitro scratch assay was performed to measure the effect of IL-1β in fibroblasts motility. Percentage of the total wound area closed by fibroblasts after 24 h with or without IL-1β (10 ng/mL). Representative images of the wound formed after the scratch (time 0 h) and 24 h after, with or without IL-1β. The dashed yellow lines show the edges of the wound. Scale bar, 100 µm. (**B**) Fold fibroblast migration through a transwell insert with or without IL-1β (10 ng/mL) in the lower compartment of the well. Representative images of migrating cells are shown at the top. (**C**) Effect of IL-1β (10 ng/mL) in fibroblast cell density. Fibroblasts were cultured with or without IL-1β for 72 h, and cell numbers were determined by counting nuclei stained with DAPI. The horizontal dashed line corresponds to the untreated fold expression (ratio = 1). (**D**) Fibroblast proliferation under IL-1β was evaluated by using the Click-iT Edu flow cytometry assay kit. Results are shown as % of Click-it + cells (proliferating cells) with respect to total cells. (**E**) Effect of proliferation inhibition in fibroblasts abilities to close a gap in a scratch assay, which was performed as in (**A**) by adding the proliferation inhibitor Mitomycin C (5 µg/mL) (Mit C) after wounding. Representative images at 0 and after 24 h with/without Mit C are shown on the Left, Scale bar as in (**A**). Quantification of the percentage of the total wound area closed is shown on the Right. Statistical analysis as in Figure 1, * *p* < 0.05, ** *p* < 0.01, *** *p* < 0.005.

To examine the potential functional impact of the mechanical and cytoskeletal changes elicited by IL-1β on pulmonary fibroblasts in the context of repair, we used the well-established scratch assay that is frequently applied to assess wound-repair in culture [42]. Of note, IL-1β significantly abrogated the ability of pulmonary fibroblasts from randomly selected donors (*n* = 6) to refill the gap inflicted on the cell monolayer upon scratch compared to untreated fibroblasts (Figure 4A). To assess whether such impaired wound-repair could be due to altered migration and/or proliferation, we first used the standard Boyden chamber migration assay and found that IL-1β enhanced the migration of pulmonary fibroblasts compared to control (Figure 4B and Supplementary Figure S2). In contrast, stimulating fibroblasts with IL-1β for three days significantly reduced their cell number (Figure 4C) concomitantly with a downregulation in cell proliferation, as indicated by Click-iT flow cytometry measurements (Figure 4D). To further confirm the negative impact of fibroblast proliferation in wound-repair, we repeated the scratch assay in the absence of IL-1β, with the addition of the mitosis inhibitor Mitomycin C (Mit C), and found that it significantly reduced the filled wound area compared to untreated fibroblasts from randomly selected donors (*n* = 6) (Figure 4E), mimicking the reduction obtained by IL-1β (Figure 4A). These results reveal that the reduction in fibroblast contractility elicited by IL-1β had mixed direct effects on repair-associated functions since it enhanced migration while it decreased proliferation. Moreover, the latter effect appears to be a major contributor to the attenuated ability of fibroblasts to refill the gap in a scratch assay.

## 3. Discussion

Normal pulmonary functions are intimately associated with the maintenance of lung-specific tissue elasticity. Such elasticity may become transiently compromised in response to lung damage as part of repair processes, whereas it becomes permanently altered in severe lung pathologies, including pulmonary fibrosis, lung cancer, and emphysema. IL-1β is a prominent cytokine of the pro-inflammatory IL-1 family that is not generally expressed in healthy tissue cells, whereas it becomes rapidly induced by pathogen products or factors released by damaged cells to orchestrate tissue early responses to damage in the lung and other organs [8,18,24]. Because fibroblasts have been identified as a key cell type in controlling mechanical tissue homeostasis under normal and diseased conditions [8], they are expected to modulate their mechanical properties in response to pro-inflammatory cytokines like IL-1β. However, how IL-1β impacts the mechanical performance of pulmonary fibroblasts has remained unclear [28,30].

In this study, we used AFM to probe the submicroscopic stiffness of primary human pulmonary fibroblasts in response to IL-1β and found that it elicited a marked decrease in cell stiffness. To our knowledge, this is the first AFM study of the nanomechanical effects of IL-1β on fibroblasts. Because AFM is a surface technique, we were limited to probing fibroblasts cultured in two-dimensional (2D) culture substrata rather than in 3D ECM gels to render the top fibroblast surface accessible to the AFM probe. Yet, in agreement with our findings, an early microscopic softening was observed in 3D type I collagen gels populated with the human pulmonary fibroblast line LL-24, as assessed by magnetic twisting cytometry [19]. Likewise, IL-1β was found to inhibit the macroscopic contractility of 3D collagen gels populated with rat pulmonary fibroblasts [22]. Similarly, IL-1β elicited a macroscopic decrease in gel stiffness in a bioartificial tendon model based on a tenocyte-populated type I collagen gel [29]. In contrast, IL-1β was found to increase the stiffness of chondrocytes as measured by optical magnetic twisting cytometry [28], which are key mesenchymal cells underlying the mechanical homeostasis of the cartilage [43]. Therefore, our results provide the first direct evidence of the mechanical impact of IL-1β on pulmonary fibroblasts in terms of reduced stiffness, and collectively support that the mechanical effects of IL-1β on mesenchymal cells are not universal but rather cell-type dependent [28].

We and others have shown that disrupting the integrity of the actomyosin CSK by inhibiting actin polymerization or myosin activity reduces the stiffness of pulmonary fibroblasts and other lung cell types, as measured by AFM and other techniques [14,32,44]. Consistently, we found that the IL-1β-induced fibroblast softening was concurrent with a reduction in F-actin as well as its assembly into stress fibers, which are frequently associated with enhanced mechanical tension [37,38]. Moreover, we also observed a reduction in α-SMA expression upon IL-1β stimulation, which is a major CSK protein underlying the enhanced contractility of TGF-β1-activated fibroblasts as frequently observed at the late stages of canonical wound repair [8,17,18,20]. In line with our observations, previous studies reported that IL-1β downregulated α-SMA in pulmonary fibroblasts and vascular smooth muscle cells [19,22,30,45]. In contrast, IL-1β increased stress fiber formation in chondrocytes and endothelial cells [27,28]. These results underscore further that the impact of IL-1β in the mechanics and the actin CSK of mesenchymal cells depends on their specific lineage [28], which is intriguing considering that activated fibroblasts in culture are known to give rise to other mesenchymal lineages, including chondrocyte-like cells, adipocytes, and endothelial cells when subjected to appropriate stimuli [6]. The association between IL-1β and the remodeling of the actomyosin CSK was not surprising since there is evidence that IL-1β and its closely related pro-inflammatory cytokine IL-1α regulate the activity of the Rho family of small GTPases, which includes key regulators of the actin CSK [21]. However, how IL-1β signals to either increase or decrease actin stress fibers and contractility in a cell type-specific fashion remains to be elucidated.

Lung architecture and mechanics are strongly influenced by the turnover of fibrillar type I and type III collagens [12], which is regulated by several factors, including the expression of *COL1A1* and *COL3A1* genes as well as that of their major degrading enzymes such as *MMP1* and *MMP2*. We found that IL-1β downregulated the mRNA levels of *COL1A1,* whereas it upregulated those of *COL3A1* as well as two important secreted collagenases in pulmonary fibroblasts, eliciting a high *COL3A1/COL1A1* ratio and strongly favoring the net degradation of type I collagen. Similar results were reported in 2D cultures of the normal human fibroblast line CCD-19Lu [30] and in 3D collagen gels of the pulmonary fibroblast line LL-24 [19]. In agreement with our findings, a dramatic decrease in type I collagen by IL-1β was reported in cultured cardiac fibroblasts [46] and in 3D collagen gels populated with tenocytes [29]. Of note, an inverse transcriptional regulation between *COL1A1* and *MMP1* has been extensively reported [9,47], and our results reveal that such inverse relation can be extended to IL-1β signaling. On the other hand, the positive impact of IL-1β on the mRNA levels of *MMP1* and *MMP2* is consistent with the general enhancement of MMP expression by IL-1β in fibroblasts reported elsewhere [30]. Collectively, our results support that IL-1β may reduce local tissue rigidity by acting both intracellularly and extracellularly through the downregulation of fibroblast stiffness and type I collagen deposition, respectively. In contrast, the mechanical and functional consequences of the opposing effects of IL-1β on fibrillar collagens observed here and elsewhere (i.e., increase in *COL3A1* and decrease in *COL1A1*) are currently undefined. However, it is worth noting that an increased collagen III:I ratio is a hallmark of fetal wound healing [48], and has been associated with the unique ability of fetal tissues to heal without scar formation, unlike adult tissues [40].

The physiopathological implications of the altered mechanobiology elicited by IL-1β on pulmonary fibroblasts in the context of lung repair reported in this study are currently unclear. However, our results suggest that the IL-1β-dependent fibroblast softening may play both direct and indirect repair-associated functions. Direct functions are associated with the enhanced migration, for it may facilitate drawing fibroblasts at the site of tissue damage. Intriguingly, such increased migration was not sufficient to counterbalance the inhibitory effects of IL-1β on proliferation, which appeared to be a major contributor to the attenuated fibroblast ability to refill the gap in a scratch assay upon IL-1β stimulation. Consistent antiproliferative effects of IL-1β on pulmonary fibroblasts have been reported elsewhere [22]. In contrast, there is conflicting literature on the role of IL-1β on fibroblast migration, with some studies reporting either increased or decreased migration in fibroblasts and fibroblast-like cells [49,50,51]. However, it is worth noting that our observed association between IL-1β-dependent fibroblast softening and enhanced migration is consistent with previous studies of cancer cell mechanics reporting a strong correlation between cell softening and increased ability to migrate through physical constraints [52,53], supporting a general trend between cell softening and enhanced migration.

In addition to direct repair-associated effects, we can envision that the mechanical alterations elicited by IL-1β on pulmonary fibroblasts may modulate lung repair indirectly through at least two complementary processes. Firstly, by increasing local pulmonary tissue compliance to facilitate normal lung distension and function [4], as suggested by the IL-1β-dependent reduction in cell contractility and deposition of type I collagen. In agreement with this notion, IL-1β increased the compliance and maximum strain in a bioartificial tendon model, which was interpreted as a protective mechanical event to prevent tendon rupture caused by excessive strain [29]. Secondly, by limiting or delaying fibrosis, at least during the early stages of tissue repair. Thus, a hallmark of fibrosis in the lung and other organs is the persistent accumulation of hypercontractile (α-SMA positive) fibroblasts in the background of excessive deposition of type I collagen, which elicits an abnormally stiff microenvironment that promotes fibrosis expansion further [9,20,54]. In contrast, IL-1β reduced the number and contractility of fibroblasts while favoring a reduction in type I collagen deposition. Indeed, we found that IL-1β downregulated all standard fibroblast activation markers examined (i.e., F-actin, α-SMA, and *COL1A1*) except *COL3A1*. Moreover, a previous study reported that matrix rigidity amplifies pulmonary fibrosis through a reduction in COX-2 signaling in pulmonary fibroblasts [9], whereas IL-1β increases COX-2 as reported here and elsewhere [35,55]. Nonetheless, it is worth reminding that IL-1β has been negatively implicated with pulmonary fibrosis as part of the pathologic role of chronic inflammation [23,24]. Yet, the pro-fibrotic effects of IL-1β have been associated with an aberrant sustained IL-1β signaling, possibly due to repeated injury, as well as with pathologic crosstalk with pro-fibrotic cytokines like TGF-β1 [19,30], which tend to accumulate at later stages of tissue response to damage [8,17,18]. Therefore, our observations suggest that the potential reparative and/or anti-fibrotic effects of the mechanobiology impact of IL-1β in pulmonary fibroblasts may be transient and limited to earlier stages of repair responses. It remains to be determined the fibroblast mechanobiology impact of pro-inflammatory factors other than IL-1β, and whether they act synergistically, and how the impact of pro-inflammatory factors are modulated by other microenvironmental cues that may become altered during early repair responses.

In summary, our results shed light on the altered mechanobiology of healthy pulmonary fibroblasts by IL-1β and its potential impact on lung repair. These observations may also help current general research efforts for directing inflammation towards pro-repair rather than pro-fibrotic processes [8].

## 4. Materials and Methods

### 4.1. Primary Culture of Human Pulmonary Fibroblasts

Primary fibroblasts were isolated in sterile conditions by an outgrowth of tissue explants from pulmonary tissue of donors with no history of pulmonary disease who were undergoing surgical treatment for spontaneous pneumothorax (*n* = 10), as described elsewhere [35]. No histopathological evidence of disease was found in these tissue samples. Donor patients were Caucasic, young (30.7 ± 7.3 y.o.), and mostly male (80%). Detailed clinical characteristics are shown in Table 1. Written informed consent was obtained from all patients according to institutional guidelines, and the study was approved by the Ethics Committee of the Hospital Clínic. Primary fibroblasts were cultured in Dulbecco’s modified Eagle’s media (DMEM) (Lonza, Basel, Switzerland) supplemented with 10% fetal bovine serum (FBS), 100 IU/mL penicillin, 100 mg/mL streptomycin (Invitrogen, Carlsbad, CA, USA), and 2 mg/mL amphotericin B (Sigma, St. Louis, MO, USA). Cultures were kept in a 5% CO_2_ humidified atmosphere at 37 °C. Fibroblasts were grown to subconfluence and subcultured by 0.05% trypsin-0.02% EDTA treatment and used up to passage five to six to prevent replicative senescence. Unless otherwise indicated, the culture medium was removed when cells reached 80% confluence and replaced by serum-free medium (SFM) for 24 h to arrest them, and kept in SFM in the absence or presence of 10 ng/mL IL-1β (R and D systems) for 4, 24 (for Western blot measurements) or 72 h (for qRT-PCR, immunofluorescence, and AFM nanoindentation measurements).

### 4.2. AFM Nanoindentation Measurements

Single fibroblast stiffness was assessed from nanoindentation measurements with AFM, as reported in [15]. In brief, a stand-alone AFM (Bioscope, Veeco, Plainview, NY, USA) was adapted to an optical microscope and provided with a low-spring constant (0.03 nN/nm) AFM cantilever (Veeco) was used to assess Young’s modulus (*E*) of single fibroblasts. For this purpose, three force versus displacement curves (F vs. z) were recorded on the perinuclear region of each fibroblast at a moderate loading force (<1 nN) and low speed (~5 µm/s). *E* of single fibroblasts was computed by fitting a suitable contact elastic model to each F vs. z curve and averaging it over three recordings. Final *E* for a fibroblast population for each donor and experimental condition was calculated from at least nine measurements as described [32].

### 4.3. qRT-PCR

For the transcriptional analysis of collagen genes, total RNA was isolated using the RNeasy mini kit (Qiagen, Hilden, Germany) according to the instructions of the manufacturer. RNA was reverse-transcribed into cDNA with the High Capacity cDNA Reverse Transcriptase kit with RNase Inhibitor (Applied Biosystems, Foster City, CA, USA). cDNA was amplified by real-time PCR (7900 HT Fast Real-Time PCR System, Applied Biosystems) using TaqMan Gene Expression Assays (Applied Biosystems) for collagen type 1, alpha 1 (*COL1A1*; Hs00164004_m1), collagen type 3, alpha 1 (*COL3A1*; Hs00943809_m1) and RNA polymerase II, polypeptide A (*RPII*; Hs00172187_m1) as an endogenous gene. For MMPs expression analysis, RNA was isolated using Ezna Total RNA Purification Kit 1 (VWR) following the manufacturer’s instructions. cDNA was generated using The PrimeScript RT Reagent Kit (Perfect Real Time) (Takara Bio Inc., Kusatsu, Japan), and real-time PCR was performed by the iQTM5 Multicolor RT-PCR Detection System (Bio-Rad, Hercules, CA, USA) with One Step TB Green PrimeScript RT-PCR Kit (Takara Bio Inc.). *ACTB* was used as an endogenous gene. The specific primers were (5′ to 3′): *ACTB*-forward: GACCCAGATCATGTTTGAGA; *ACTB*-reverse: AGGGCATACCCCTCGTAGAT; *MMP1*-forward: AAATCCTGTCCAGCCCATCG, *MMP1*-reverse: GCAGTTGTGGCCAGAAAACA; *MMP2*-forward: CCGATGGGGAGTACTGCAAG and *MMP2*-reverse: GCGGAATGGAAACTTGCAGG. Gene expression data were calculated using the 2^−ΔCt^ method [56], and the results expressed as fold-change with respect to untreated control samples.

### 4.4. Western Blotting

Cells were lysed with RIPA buffer (TrisHCl 50 mM, NaCl 150 mM (pH 7,4), 1% NP40 Igepal, 1% Triton X-100, 0.1% SDS and 5 µL/mL of a protease inhibitor cocktail (Sigma)). 12.5 to 30 ng of protein cells were loaded on 7% Tris-Acetate gels and run (125 V, 90 min) in a Novex XCell II Mini-Cell (Invitrogen). Proteins were transferred to a nitrocellulose membrane and blocked using 5% non-fat dry milk, 0.1% Tween 20 in PBS. Membranes were incubated overnight with primary antibodies against COX-2 (Santa Cruz Biotechnology, Dallas, TX, USA), α-SMA (Sigma), or β-actin (Sigma) and subsequently incubated with an appropriate peroxidase-labeled secondary antibody. Protein bands were visualized by chemiluminescence (LAS3000, Fujifilm, Tokyo, Japan), and densitometry analysis was performed by Image gauge v.4.0 software (Fuji Photo Film Co., Ltd., Tokyo, Japan).

### 4.5. Immunofluorescence

Cells grown in 4-well CultureSlides were fixed with cold 4% paraformaldehyde for 15 min and permeabilized with 0.5% Triton X-100 for 30 min. Once blocked with 1% bovine serum albumin-PBS for 1 h, a primary antibody against α-SMA (Sigma, 1:500) was added for 1 h at 37 °C. A suitable fluorescent secondary antibody and Phalloidin-TRITC (Sigma) to detect F-actin were also incubated for 1 h. Cells were counterstained with DAPI (1:10.000) and mounted with Prolong^TM^ Gold antifade reagent (Thermo Fisher Scientific, Waltham, MA, USA). Epifluorescence microscopy (Leica Microsystems, Wetzlar, Germany) was used to image the immunostainings with a 20× objective. Processing and analysis of immunofluorescence images were carried out with ImageJ/Fiji software [57]. F-actin and α-SMA images were background subtracted and used to compute the total intensity, which was normalized by the corresponding total cell number and averaged for each patient and condition.

### 4.6. Cell Proliferation Assay

The proliferative status of fibroblasts was analyzed using the Click-iT EdU Alexa Fluor 488 Flow Cytometry Assay Kit (Invitrogen) according to the manufacturer’s instructions. Briefly, cultured fibroblasts were treated with or without IL-1β (10 ng/mL) for 24 h before the addition of the nucleoside analog EdU (5-etinil-2´-deoxiuridin), which is incorporated into the DNA only by cells under replication. Proliferative cells were evaluated using a FACSCanto II Cytometer (BS Biosciences) and BD FACSDiva software v.6.0 software (San Jose, CA, USA). Results are shown as % Click-iT^®^ positive cells (proliferating cells) with respect to total cells.

### 4.7. Migration Assay

Fibroblasts migration was performed using the standard Boyden chamber migration assay with Transwell culture plates with filter-inserts (24 Transwell plate, 8 µm filter pore size; Merck Millipore, Burlington, MA, USA). Fibroblasts were seeded on the filter-inserts at 3 × 10^4^ cells/cm^2^ in SFM. SFM with or without IL-1β (10 ng/mL) was added to the lower Transwell compartment. After 16 h, cells were fixed with 100% methanol for 15 min and incubated with 0.5% crystal violet (Sigma) for 30 min. Cells that had migrated into the lower side of the filter were imaged by a phase-contrast microscope provided with a color camera. Migration was normalized to that obtained in the control medium. All image processing was conducted with ImageJ.

### 4.8. In Vitro Scratch Wound Assay

Fibroblasts were seeded in 6-well plates and grown until confluence. A scratch was made on the cell monolayer using a pipette tip, and wound closure was monitored by acquiring bright-field images at 0 h and 24 h with or without 10 ng/mL IL-1β in 1% FBS-DMEM medium. In some experiments, the proliferation inhibitor Mitomycin C (5µg/mL) (Sigma) was added after wounding. The percentage of the closed area was evaluated by ImageJ (v.1.51j8).

### 4.9. Statistical Analysis

Two-group comparisons were carried out using Student’s *t*-test (Graphpad software, v5.01, San Diego, CA, USA). Statistical significance was assumed at *p* < 0.05. Data are given as mean ± standard error (SE).

## Figures and Tables

**Table 1:** Basic clinical characteristics of our donor cohort.

Patient Ref.	Gender	Age	Race
#150	Male	26	Caucasian
#166	Male	35	Caucasian
#169	Male	38	Caucasian
#170	Female	34	Caucasian
#177	Male	20	Caucasian
#180	Male	31	Caucasian
#186	Male	37	Caucasian
#195	Female	36	Caucasian
#197	Male	17	Caucasian
#200	Male	33	Caucasian

## Supplementary Materials

The following are available online at https://www.mdpi.com/1422-0067/21/22/8417/s1, Figure S1: Expression of fibrillar collagens in primary lung fibroblasts upon stimulation with IL-1β (10 ng/mL) for 72 h. Figure S2: Fibroblasts migration upon stimulation with IL-1β is seeding density independent.

## Abbreviations

IL-1β	Interleukin-1β
ECM	extracellular matrix
TGF-β	transforming growth factor-beta
*E*	Young´s elastic modulus
AFM	Atomic force microscopy
COX-2	cyclooxygenase-2
CSK	cytoskeleton
F-actin	filamentous actin
α-SMA	alpha-smooth muscle actin
MMP-1	matrix metalloproteinase-1
MMP-2	matrix metalloproteinase-2
Mit C	Mitomycin C
DMEM	Dulbecco’s modified Eagle’s media
FBS	fetal bovine serum
SFM	serum-free medium
EdU	5-etinil-2´-deoxiuridin

## References

[1] DuFort C.C., Paszek M.J., Weaver V.M. (2011). Balancing forces: Architectural control of mechanotransduction. Nat. Rev. Mol. Cell Biol..

[2] Vining K.H., Mooney D.J. (2017). Mechanical forces direct stem cell behaviour in development and regeneration. Nat. Rev. Mol. Cell Biol..

[3] Burgstaller G., Oehrle B., Gerckens M., White E.S., Schiller H.B., Eickelberg O. (2017). The instructive extracellular matrix of the lung: Basic composition and alterations in chronic lung disease. Eur. Respir. J..

[4] Tschumperlin D.J., Boudreault F., Liu F. (2010). Recent advances and new opportunities in lung mechanobiology. J. Biomech..

[5] Tschumperlin D.J., Lagares D. (2020). Mechano-therapeutics: Targeting Mechanical Signaling in Fibrosis and Tumor Stroma. Pharmacol. Ther..

[6] Kalluri R. (2016). The biology and function of fibroblasts in cancer. Nat. Rev. Cancer.

[7] Mescher A.L. (2017). Macrophages and fibroblasts during inflammation and tissue repair in models of organ regeneration. Regeneration.

[8] Hannan R.T., Peirce S.M., Barker T.H. (2018). Fibroblasts: Diverse Cells Critical to Biomaterials Integration. ACS Biomater. Sci. Eng..

[9] Liu F., Mih J.D., Shea B.S., Kho A.T., Sharif A.S., Tager A.M., Tschumperlin D.J. (2010). Feedback amplification of fibrosis through matrix stiffening and COX-2 suppression. J. Cell Biol..

[10] Harris R.S. (2005). Pressure-volume curves of the respiratory system. Respir. Care.

[11] Puig M., Lugo R., Gabasa M., Gimenez A., Velasquez A., Galgoczy R., Ramirez J., Gomez-Caro A., Busnadiego O., Rodriguez-Pascual F. (2015). Matrix Stiffening and beta(1) Integrin Drive Subtype-Specific Fibroblast Accumulation in Lung Cancer. Mol. Cancer Res..

[12] Suki B., Ito S., Stamenovic D., Lutchen K.R., Ingenito E.P. (2005). Biomechanics of the lung parenchyma: Critical roles of collagen and mechanical forces. J. Appl. Physiol..

[13] Alcaraz J., Otero J., Jorba I., Navajas D. (2018). Bidirectional mechanobiology between cells and their local extracellular matrix probed by atomic force microscopy. Semin. Cell Dev. Biol..

[14] Acerbi I., Luque T., Gimenez A., Puig M., Reguart N., Farre R., Navajas D., Alcaraz J. (2012). Integrin-specific mechanoresponses to compression and extension probed by cylindrical flat-ended AFM tips in lung cells. PLoS ONE.

[15] Gabasa M., Duch P., Jorba I., Giménez A., Lugo R., Pavelescu I., Rodríguez-Pascual F., Molina-Molina M., Xaubet A., Pereda J. (2017). Epithelial contribution to the pro-fibrotic stiff microenvironment and myofibroblast population in lung fibrosis. Mol. Biol. Cell.

[16] Van Linthout S., Miteva K., Tschöpe C. (2014). Crosstalk between fibroblasts and inflammatory cells. Cardiovasc. Res..

[17] Tomasek J.J., Gabbiani G., Hinz B., Chaponnier C., Brown R.A. (2002). Myofibroblasts and mechano-regulation of connective tissue remodelling. Nat. Rev. Mol. Cell Biol..

[18] Saxena A., Chen W., Su Y., Rai V., Uche O.U., Li N., Frangogiannis N.G. (2013). IL-1 induces proinflammatory leukocyte infiltration and regulates fibroblast phenotype in the infarcted myocardium. J. Immunol..

[19] Leung L.Y., Tian D., Brangwynne C.P., Weitz D.A., Tschumperlin D.J. (2007). A new microrheometric approach reveals individual and cooperative roles for TGF-beta1 and IL-1beta in fibroblast-mediated stiffening of collagen gels. FASEB J..

[20] Hinz B., Phan S.H., Thannickal V.J., Prunotto M., Desmoulière A., Varga J., De Wever O., Mareel M., Gabbiani G. (2012). Recent developments in myofibroblast biology. Paradigms for connective tissue remodeling. Am. J. Pathol..

[21] Lie P.P., Cheng C.Y., Mruk D.D. (2012). The biology of interleukin-1: Emerging concepts in the regulation of the actin cytoskeleton and cell junction dynamics. Cell. Mol. Life Sci. CMLS.

[22] Zhang H.Y., Gharaee-Kermani M., Phan S.H. (1997). Regulation of lung fibroblast alpha-smooth muscle actin expression, contractile phenotype, and apoptosis by IL-1beta. J. Immunol..

[23] Hoffman H.M., Wanderer A.A. (2010). Inflammasome and IL-1beta-mediated disorders. Curr. Allergy Asthma Rep..

[24] Borthwick L.A. (2016). The IL-1 cytokine family and its role in inflammation and fibrosis in the lung. Semin. Immunopathol..

[25] Parisi V., Leosco D. (2020). Precision Medicine in COVID-19: IL-1β a Potential Target. JACC. Basic Transl. Sci..

[26] Postlethwaite A.E., Raghow R., Stricklin G.P., Poppleton H., Seyer J.M., Kang A.H. (1988). Modulation of fibroblast functions by interleukin 1: Increased steady-state accumulation of type I procollagen messenger RNAs and stimulation of other functions but not chemotaxis by human recombinant interleukin 1 alpha and beta. J. Cell Biol..

[27] Campos S.B., Ashworth S.L., Wean S., Hosford M., Sandoval R.M., Hallett M.A., Atkinson S.J., Molitoris B.A. (2009). Cytokine-induced F-actin reorganization in endothelial cells involves RhoA activation. Am. J. Physiol. Renal. Physiol..

[28] Chen C., Xie J., Rajappa R., Deng L., Fredberg J., Yang L. (2015). Interleukin-1β and tumor necrosis factor-α increase stiffness and impair contractile function of articular chondrocytes. Acta Biochim. Biophys. Sin..

[29] Qi J., Chi L., Maloney M., Yang X., Bynum D., Banes A.J. (2006). Interleukin-1beta increases elasticity of human bioartificial tendons. Tissue Eng..

[30] Mia M.M., Boersema M., Bank R.A. (2014). Interleukin-1β attenuates myofibroblast formation and extracellular matrix production in dermal and lung fibroblasts exposed to transforming growth factor-β1. PLoS ONE.

[31] Li M., Dang D., Liu L., Xi N., Wang Y. (2017). Atomic Force Microscopy in Characterizing Cell Mechanics for Biomedical Applications: A Review. IEEE Trans. Nanobiosci..

[32] Alcaraz J., Xu R., Mori H., Nelson C.M., Mroue R., Spencer V.A., Brownfield D., Radisky D.C., Bustamante C., Bissell M.J. (2008). Laminin and biomimetic extracellular elasticity enhance functional differentiation in mammary epithelia. EMBO J..

[33] Viji Babu P.K., Rianna C., Belge G., Mirastschijski U., Radmacher M. (2018). Mechanical and migratory properties of normal, scar, and Dupuytren’s fibroblasts. J. Mol. Recognit..

[34] Stylianou A., Kontomaris S.V., Grant C., Alexandratou E. (2019). Atomic Force Microscopy on Biological Materials Related to Pathological Conditions. Scanning.

[35] Gabasa M., Royo D., Molina-Molina M., Roca-Ferrer J., Pujols L., Picado C., Xaubet A., Pereda J. (2013). Lung Myofibroblasts Are Characterized by Down-Regulated Cyclooxygenase-2 and Its Main Metabolite, Prostaglandin E2. PLoS ONE.

[36] Roca-Ferrer J., Garcia-Garcia F.J., Pereda J., Perez-Gonzalez M., Pujols L., Alobid I., Mullol J., Picado C. (2011). Reduced expression of COXs and production of prostaglandin E(2) in patients with nasal polyps with or without aspirin-intolerant asthma. J. Allergy Clin. Immunol..

[37] Roca-Cusachs P., Alcaraz J., Sunyer R., Samitier J., Farre R., Navajas D. (2008). Micropatterning of single endothelial cell shape reveals a tight coupling between nuclear volume in G1 and proliferation. Biophys. J..

[38] Kumar S., Maxwell I.Z., Heisterkamp A., Polte T.R., Lele T.P., Salanga M., Mazur E., Ingber D.E. (2006). Viscoelastic retraction of single living stress fibers and its impact on cell shape, cytoskeletal organization, and extracellular matrix mechanics. Biophys. J..

[39] Bonnans C., Chou J., Werb Z. (2014). Remodelling the extracellular matrix in development and disease. Nat. Rev. Mol. Cell Biol..

[40] Namazi M.R., Fallahzadeh M.K., Schwartz R.A. (2011). Strategies for prevention of scars: What can we learn from fetal skin?. Int. J. Dermatol..

[41] Pardo A., Selman M. (2012). Role of matrix metaloproteases in idiopathic pulmonary fibrosis. Fibrogenes. Tissue Repair.

[42] Pomari E., Dalla Valle L., Pertile P., Colombo L., Thornton M.J. (2015). Intracrine sex steroid synthesis and signaling in human epidermal keratinocytes and dermal fibroblasts. FASEB J..

[43] Akkiraju H., Nohe A. (2015). Role of Chondrocytes in Cartilage Formation, Progression of Osteoarthritis and Cartilage Regeneration. J. Dev. Biol..

[44] Gavara N., Sunyer R., Roca-Cusachs P., Farré R., Rotger M., Navajas D. (2006). Thrombin-induced contraction in alveolar epithelial cells probed by traction microscopy. J. Appl. Physiol..

[45] Kuang P.P., Berk J.L., Rishikof D.C., Foster J.A., Humphries D.E., Ricupero D.A., Goldstein R.H. (2002). NF-kappaB induced by IL-1beta inhibits elastin transcription and myofibroblast phenotype. Am. J. Physiol. Cell Physiol..

[46] Xiao H., Ji A.M., Li Z.L., Song X.D., Su D., Chen A.H. (2008). Interleukin-1β inhibits collagen synthesis and promotes its decomposition in cultured cardiac fibroblasts. Sheng Li Xue Bao Acta Physiol. Sin..

[47] Gimenez A., Duch P., Puig M., Gabasa M., Xaubet A., Alcaraz J. (2017). Dysregulated Collagen Homeostasis by Matrix Stiffening and TGF-beta1 in Fibroblasts from Idiopathic Pulmonary Fibrosis Patients: Role of FAK/Akt. Int. J. Mol. Sci..

[48] Hallock G.G., Merkel J.R., Rice D.C., DiPaolo B.R. (1993). The ontogenetic transition of collagen deposition in rat skin. Ann. Plast. Surg..

[49] Mitchell M.D., Laird R.E., Brown R.D., Long C.S. (2007). IL-1beta stimulates rat cardiac fibroblast migration via MAP kinase pathways. Am. J. Physiol. Heart Circ. Physiol..

[50] Kohyama T., Liu X., Wen F.Q., Kobayashi T., Fang Q., Abe S., Cieslinski L., Barnette M.S., Rennard S.I. (2004). Cytokines modulate cilomilast response in lung fibroblasts. Clin. Immunol..

[51] Cao C., Wu F., Niu X., Hu X., Cheng J., Zhang Y., Li C., Duan X., Fu X., Zhang J. (2020). Cadherin-11 cooperates with inflammatory factors to promote the migration and invasion of fibroblast-like synoviocytes in pigmented villonodular synovitis. Theranostics.

[52] Rianna C., Radmacher M., Kumar S. (2020). Direct evidence that tumor cells soften when navigating confined spaces. Mol. Biol. Cell.

[53] Rudzka D.A., Spennati G., McGarry D.J., Chim Y.H., Neilson M., Ptak A., Munro J., Kalna G., Hedley A., Moralli D. (2019). Migration through physical constraints is enabled by MAPK-induced cell softening via actin cytoskeleton re-organization. J. Cell Sci..

[54] Ho Y.Y., Lagares D., Tager A.M., Kapoor M. (2014). Fibrosis-a lethal component of systemic sclerosis. Nat. Rev. Rheumatol..

[55] Machado-Carvalho L., Martín M., Torres R., Gabasa M., Alobid I., Mullol J., Pujols L., Roca-Ferrer J., Picado C. (2016). Low E-prostanoid 2 receptor levels and deficient induction of the IL-1β/IL-1 type I receptor/COX-2 pathway: Vicious circle in patients with aspirin-exacerbated respiratory disease. J. Allergy Clin. Immunol..

[56] Schmittgen T.D., Livak K.J. (2008). Analyzing real-time PCR data by the comparative C(T) method. Nat. Protoc..

[57] Schindelin J., Arganda-Carreras I., Frise E., Kaynig V., Longair M., Pietzsch T., Preibisch S., Rueden C., Saalfeld S., Schmid B. (2012). Fiji: An open-source platform for biological-image analysis. Nat. Methods.

