# Supplementary figures and images for: Interleukin-1β Modulation of the Mechanobiology of Primary Human Pulmonary Fibroblasts: Potential Implications in Lung Repair
Source: Int J Mol Sci. 2020 Nov 10;21(22):8417. doi: 10.3390/ijms21228417 (PMC7696791; doi:10.3390/ijms21228417)

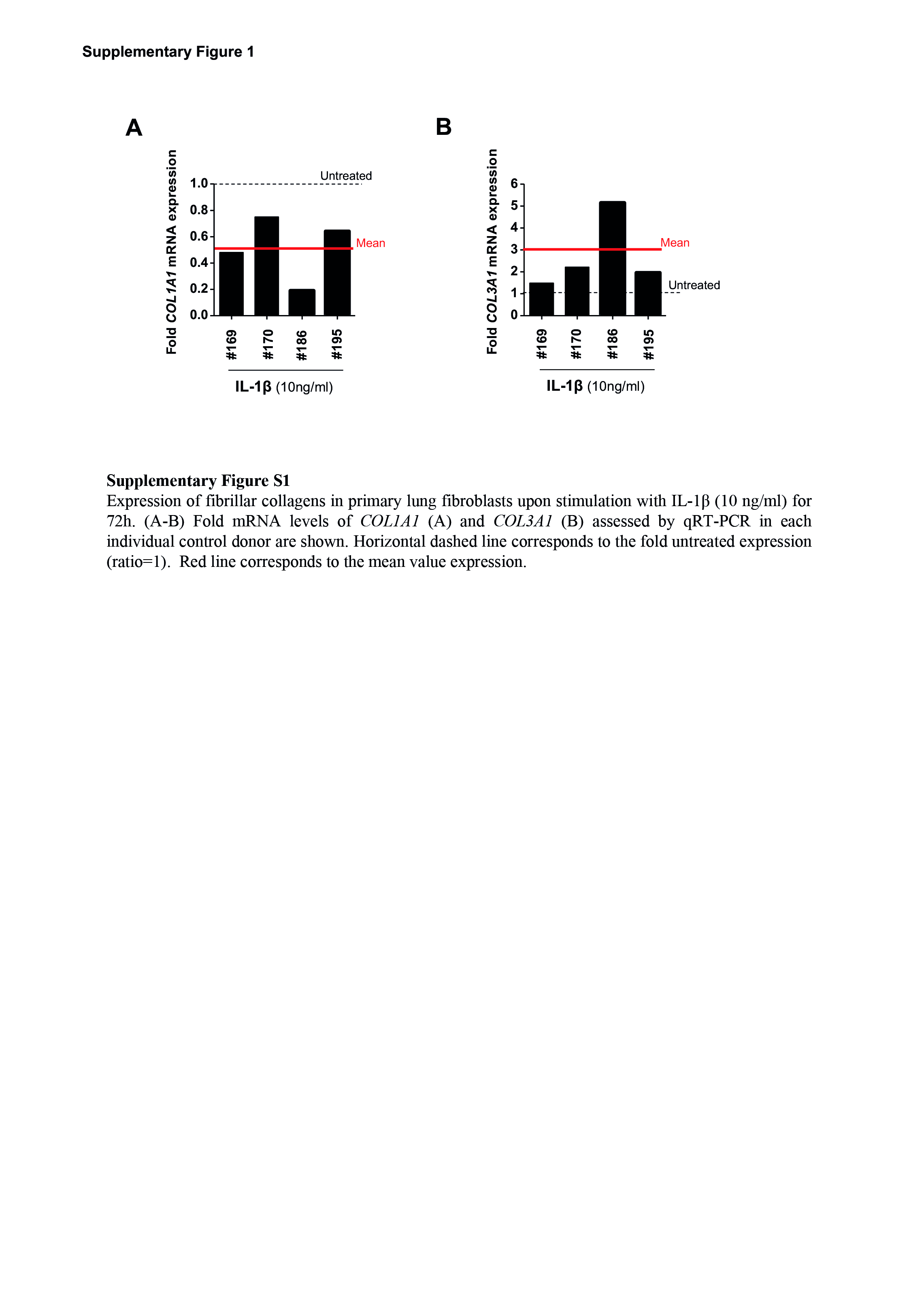

Supplement: Supplementary file 1 [file ijms-21-08417-s001.zip › supplementary fig.1.tif]

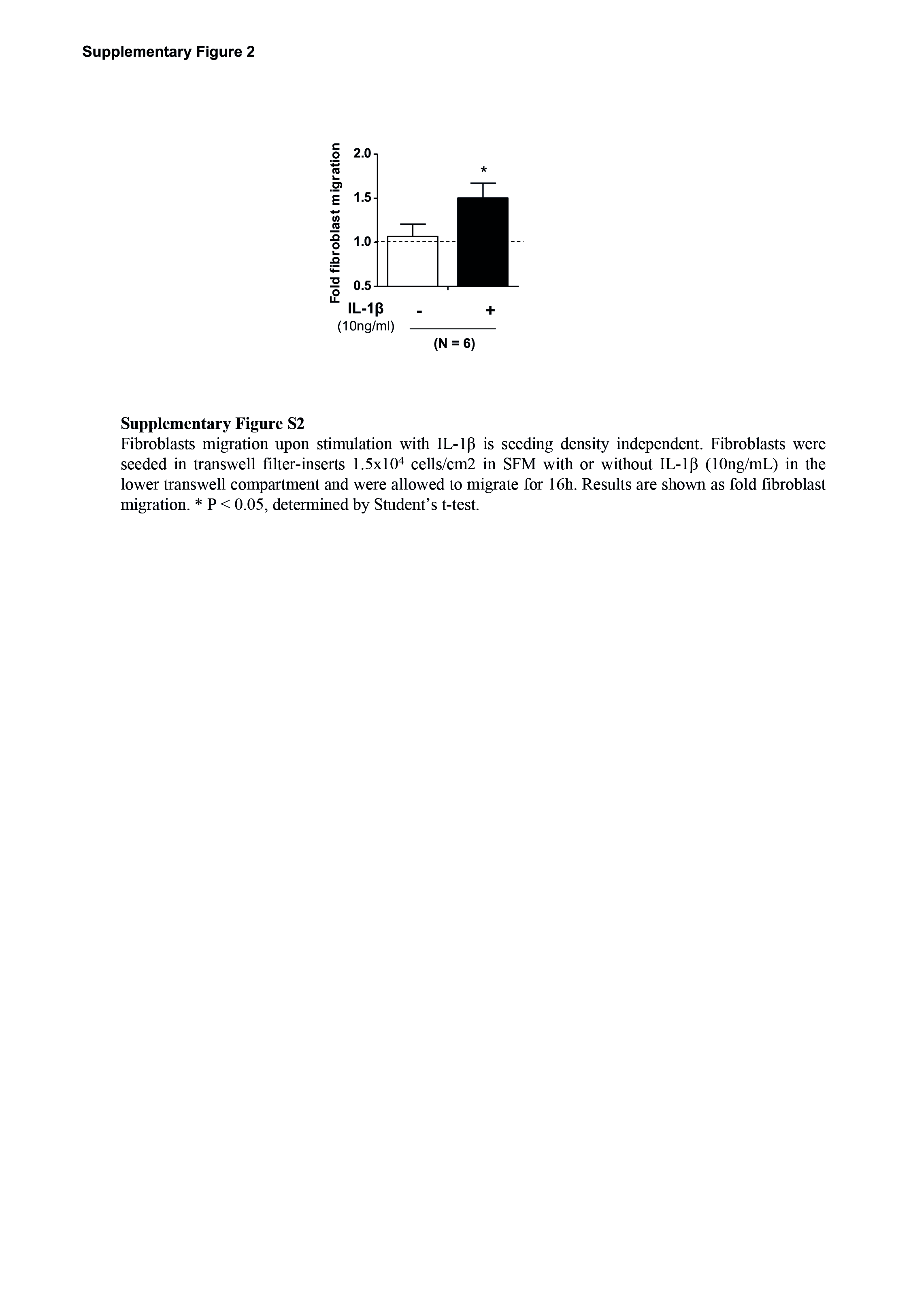

Supplement: Supplementary file 1 [file ijms-21-08417-s001.zip › supplementary fig.2.tif]
